# Is the Geographic Range of Mangrove Forests in the Conterminous United States Really Expanding?

**DOI:** 10.3390/s16122010

**Published:** 2016-11-28

**Authors:** Chandra Giri, Jordan Long

**Affiliations:** 1 Sensing and Spatial Analysis Branch, Office of Research and Development, United States Environmental Protection Agency, 109 T.W. Alexander Drive, Research Triangle Park, Durham, NC 27709, USA; 2 ARSC Research and Technology Solutions, Contractor to U.S. Geological Survey (USGS) Earth Resources Observation and Science (EROS) Center, Sioux Falls, SD 57198, USA; jordan.long.ctr@usgs.gov

**Keywords:** geographic range expansion, mangrove forests, Landsat, climate change, mangrove change

## Abstract

Changes in the distribution and abundance of mangrove species within and outside of their historic geographic range can have profound consequences in the provision of ecosystem goods and services they provide. Mangroves in the conterminous United States (CONUS) are believed to be expanding poleward (north) due to decreases in the frequency and severity of extreme cold events, while sea level rise is a factor often implicated in the landward expansion of mangroves locally. We used ~35 years of satellite imagery and in situ observations for CONUS and report that: (i) poleward expansion of mangrove forest is inconclusive, and may have stalled for now, and (ii) landward expansion is actively occurring within the historical northernmost limit. We revealed that the northernmost latitudinal limit of mangrove forests along the east and west coasts of Florida, in addition to Louisiana and Texas has not systematically expanded toward the pole. Mangrove area, however, expanded by 4.3% from 1980 to 2015 within the historic northernmost boundary, with the highest percentage of change in Texas and southern Florida. Several confounding factors such as sea level rise, absence or presence of sub-freezing temperatures, land use change, impoundment/dredging, changing hydrology, fire, storm, sedimentation and erosion, and mangrove planting are responsible for the change. Besides, sea level rise, relatively milder winters and the absence of sub-freezing temperatures in recent decades may be enabling the expansion locally. The results highlight the complex set of forcings acting on the northerly extent of mangroves and emphasize the need for long-term monitoring as this system increases in importance as a means to adapt to rising oceans and mitigate the effects of increased atmospheric CO_2_.

## 1. Introduction

**Figure 1:**
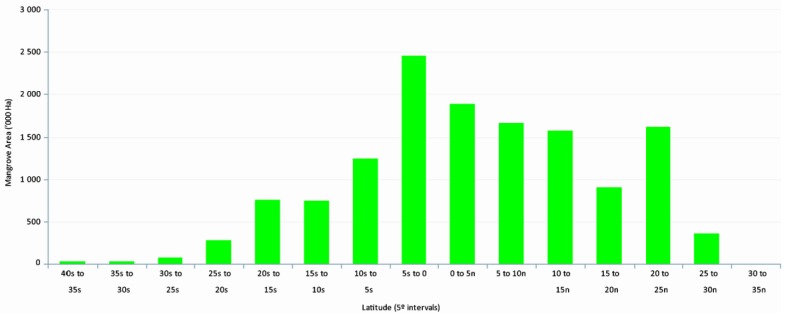
Latitudinal distribution of global mangroves [1].

Mangrove forests, consisting of multiple taxa of tropical macrophytes, are distributed mainly in tropical and subtropical regions of the world [1,2,3]. The upper latitudinal limits of global distribution, extending into the temperate regions, are characterized by decreased abundance, reduced species diversity, and decreased tree vigor, growth, and biomass (Figure 1). The geographic range of mangrove forests is highly dynamic, often expanding and contracting over time [1,4,5,6]. The range mobility could serve as an indicator of climate change reflecting changing environmental conditions or gradual niche evolution over time. What causes areas of mangrove habitat to expand or contract is an important research question with many science and policy implications [7,8]. Researchers have been interested in whether mangroves can adapt to relative sea level rise or can withstand and recover from more frequent and extreme storms for many years [9,10].

**Figure 2:**
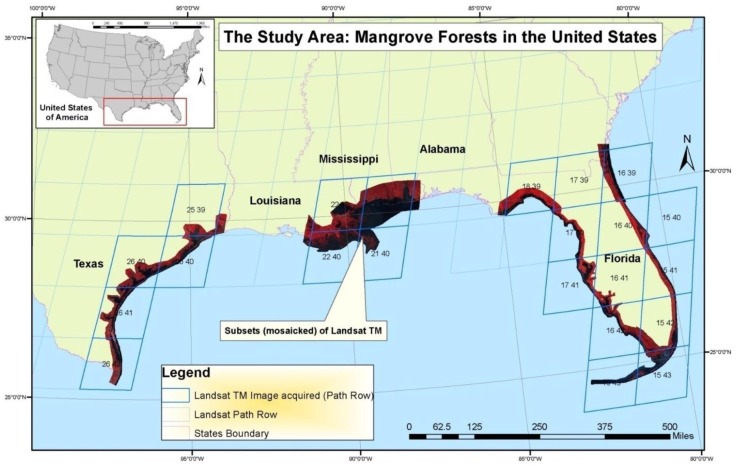
Approximate location of mangrove distribution in the conterminous United States with a subset of Landsat Thematic Mapper imagery and Landsat path/rows.

Mangrove forests of the conterminous United States (CONUS), located solely in the Gulf of Mexico states of Texas, Louisiana, and Florida (Figure 2), are believed to be expanding towards the pole due to climate change [11,12,13]. Warmer winter air temperatures and less extreme sub-freezing winter air temperatures are likely causes of the expansion of black mangrove (*Avicennia germinans*) into salt marshes dominated by smooth cordgrass (*Spartina alterniflora*) [14,15]. Other factors such as fire, tropical storms, sea level rise, sedimentation/erosion, changing hydrology and certain management practices are also believed to be contributing to the expansion in localized areas [6,12,16,17,18,19,20].

Surprisingly, findings of poleward movement are either based on short-term (<20 years) data records, tested in relatively small geographic areas (e.g., northeastern Florida), or not tested in the northernmost boundaries. For example, Cavanaugh et al. [11] provided strong evidence of poleward expansion of mangroves in northeastern Florida by analyzing Landsat satellite data from 1984 to 2011, in addition to minimum temperature data, attributing the observed poleward mangrove expansion to a reduction in the frequency of extreme cold events. However, Giri and Long [21] argued that the mangrove forests in eastern Florida may not be expanding toward the pole, but rather reemerging from damage incurred during sub-freezing temperatures in the winters of 1983 and 1989. Expansion was merely a window within a natural decadal-scale oscillation. Thus, Giri and Long [21] also suggested that a longer ecological time scale might be needed to study the impact of climate change on mangrove poleward expansion. Specific drivers vary spatially and temporally. In many ecosystems, species shifts are typically caused by complex, intertwining local-scale environmental and anthropogenic drivers sometimes entangled with climate [22,23]. 

A comprehensive assessment of rates, patterns, and causes of mangrove expansion and contraction covering the entire CONUS region is lacking. To address this gap, we analyzed ~35 years of satellite imagery to examine mangrove forest cover change across the entire CONUS. Our primary hypothesis was that mangrove expansion observed in Florida may be stalling in a systematic sense, and not expanding beyond its poleward limits but rather only replacing saltmarsh habitat locally. There are a number of potential reasons for either outcome, which we explore herein. Our intent is to reframe the discussion of mangrove expansion based on this mapping, and potentially identify several very solid limits to poleward expansion that need to be considered.

## 2. Methods

**Figure 3:**
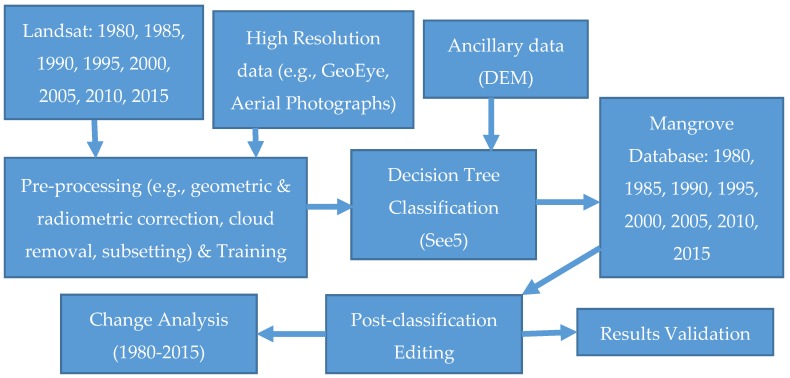
An overview of the methodology used.

Figure 3 provides an overview of the method schema for this analysis. We used Landsat, very high resolution satellite data, Aerial Photographs (AP), Digital Elevation Model (DEM), and field inventory data to map recent mangrove dynamics for CONUS. Landsat Thematic Mapper (TM), Enhanced Thematic Mapper Plus (ETM+), and Operational Land Imager (OLI) data covering both all cloud-free pixels for both summer and winter seasons were downloaded from http://glovis.usgs.gov. Aerial Photographs and very high resolution satellite data with a spatial resolution of 5 m or less were downloaded from the U.S. Geological Survey (USGS) Earth Resources Observation and Science (EROS) Center National Map Seamless Server (http://seamless.usgs.gov; restricted use), and the National Geospatial Agency (NGA) Web-based Access and Retrieval Portal (WARP) (https://warp.nga.mil/; restricted use). The Shuttle Radar Topography Mission (SRTM) 30-m DEM database was obtained from USGS EROS. Landsat data was preprocessed for geometric correction, Top-of-Atmosphere (TOA) reflectance [24], cloud and cloud shadow removal [25], and subsetting. The subsetting step involves selecting areas where mangrove forest is likely to occur and excluding areas where they are not found (i.e., far inland, highlands, and open ocean) [26], to reduce data volume that increases overall mangrove classification accuracy by reducing the spectral variation imposed by other land cover types. 

To classify the Landsat satellite data, a supervised decision tree classifier based on a univariate decision tree algorithm was used [27]. A decision tree classification is a procedure that recursively partitions a dataset into smaller subdivisions on the basis of a set of tests defined at each branch or node in the tree. The tree is composed of a root node, a set of internal nodes (splits), and a set of terminal nodes (leaves) [28]. The process of applying See5 includes constructing the training set, training the decision tree, and translating the decision tree to rules. The decision tree classification approach has several advantages over maximum likelihood classification: (1) decision trees are strictly nonparametric and do not require assumptions regarding the distributions of the input data; (2) they can handle nonlinear relations between features and classes; and (3) they can handle noisy and missing data. The nonparametric attribute is particularly important for regional and global scale studies because the broad land cover classes of interest exhibit multimodal frequency distributions [29]. The Maximum Likelihood Classifier (MLC) assumes normal distribution. Moreover, the classification accuracy of a decision tree classifier is equal to or higher than the MLC [29]. Previous research [29,30] demonstrated that boosting, a process in which the classification algorithm concentrates on those interpretation results that are more difficult to classify, consistently increases classification accuracies by 20%–50%. The decision tree classification approach is fully automated and repeatable [29].

The decision tree “ruleset” was generated using commercial software, See5 (www.rulequest.com). The output of the decision tree classification is a thematic image containing target classes: dense mangrove (mangrove canopy cover greater than 70%), sparse mangrove (mangrove canopy cover less than 70% and above 10%), open water, salt marsh/wetland (which dominates but may be mixed with a variety of rushes, sedges, and forb), barren/impervious, and other vegetation. Training areas were selected from Landsat, very high resolution satellite data such as GeoEye, aerial photographs, and other ancillary data. We mapped “true mangroves,” defined as trees and shrubs that grow exclusively in the tidal and intertidal zones [2].

We used bands 1–5 and 7 for Landsat TM and ETM+ sensors, bands 2–7 for Landsat OLI sensor, and ancillary data (e.g., DEM) as an explanatory variable and mangrove/non-mangrove classes as response variables. Seasonal mosaics were prepared by selecting best pixels. The minimum mapping unit used in this study was 0.08 ha. Following classification, additional recoding was performed to eliminate apparent classification errors. 

**Figure 4:**
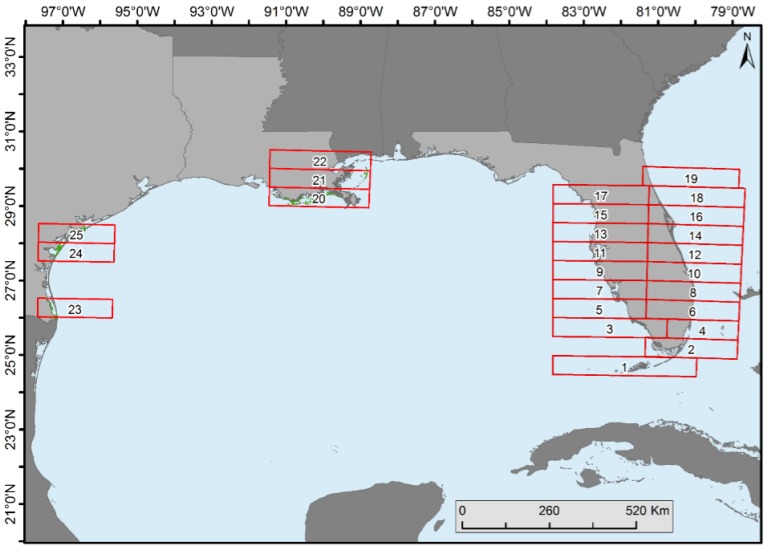
The 0.5° latitudinal zones in Florida, Louisiana, and Texas created for mangrove change analysis.

Mangrove forest cover change statistics were generated in each 0.5° latitudinal range on the east and west coasts of Florida (except in the southernmost areas where separation of east was not possible), Texas, and Louisiana (Figure 4).

Percent change was calculated as follows.

$$Percent\;Change=\frac{\mathrm{New}\;\mathrm{Value}-\mathrm{Old}\;\mathrm{Value}}{\mathrm{Old}\;\mathrm{Value}}\;\times 100$$

where, new value is a later date (e.g., 2015) and old value is an earlier date (e.g., 1980).

A two-step validation approach was adopted: qualitative and quantitative. As a qualitative check, we divided the entire area into 500 m × 500 m regularly spaced grids and checked each grid visually to identify and correct any gross errors inherent in the classification maps. Next, we generated an error matrix for 2015 classification using a validation database prepared using very high resolution imagery available in Google Earth. A total of 600 randomly selected pixels were used to prepare the validation database. These sample points were interpreted visually. Accuracy assessment of previous years was not performed because sufficient validation data were not available.

The annual mean sea level (MSL) trends and annual minimum temperature data were obtained from National Oceanic and Atmospheric Administration (NOAA) (http://tidesandcurrents.noaa.gov/sltrends/sltrends.html). These datasets were collected from gauges and stations in Grand Isle (LA, USA), Corpus Christi (TX, USA), and St. Augustine, Cedar Keys, Tampa Bay, and Naples (FL, USA). The MSL and minimum temperature data represent local trends as opposed to state, regional, or the global trends.

Linear correlation coefficient was calculated between annual minimum temperature and mangrove area change, and annual mean relative sea level rise (from tide gauges) and mangrove area change. The linear correlation coefficients were calculated using the following equation:

$$r=\frac{SS_{xy}}{\sqrt{SS_{x}SS_{y}}}$$

where

$$\begin{array}{l} SS_{x}=\sum {\left(x-\bar{x}\right)}^{2}\\ SS_{y}=\sum {\left(y-\bar{y}\right)}^{2}\\ SS_{xy}=\sum \left(x-\bar{x}\right)\left(y-\bar{y}\right) \end{array}$$

## 3. Results and Discussion

**Figure 5:**
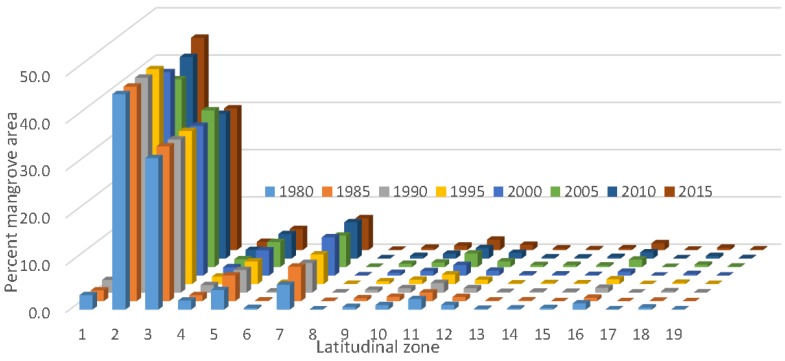
Percent mangrove forests in different latitudinal zones in Florida.

We prepared a wall-to-wall mangrove distribution and change database for CONUS every five years from 1980 to 2015. The total mangrove area in CONUS in 2015 was 251,293 ha with 98.1% of mangrove area in Florida, 0.6% in Louisiana, and 1.3% in Texas. As expected, mangrove occurrence generally decreased with increasing latitude from south to north. For example, approximately 85% of the CONUS mangrove area was found between 24.5° to 26.0° latitude. Spatial distribution percentages of Florida’s mangroves along the east and west coasts for every five years from 1980 to 2015 per 0.5° latitude are presented in Figure 5. 

**Figure 6:**
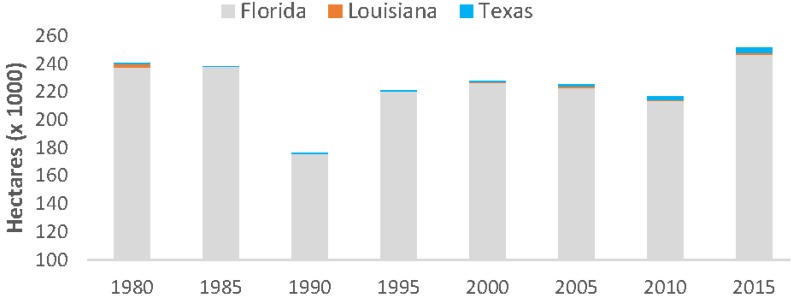
Areal extent of mangrove forests in CONUS every five years from 1980 to 2015.

Overall, mangrove forests in CONUS increased by 4.3% from 1980 to 2015; however, quinquennial variation in areal coverage fluctuated substantially and was lowest in 1990 (176,000 ha) following an extensive winter sub-freeze event in late 1989 and highest in 2015 (251,293 ha) (Figure 6). From 1980 to 2015, mangrove area increased in Florida and Texas by 3.6% and 0.9%, respectively, and decreased in Louisiana’s mangrove area by 0.2%. This is a 3.8% (9026 ha) net increase in Florida mangrove area, a 234% (2259 ha) net increase in Texas mangrove area, and a 25% (536 ha) net decrease in Louisiana’s mangrove area.

Validation of mangrove cover products based on rigorous sampling methods and high quality contemporaneous reference data is clearly desirable; however, as is very often the case, limited resources made fully rigorous quantitative validation an unreachable ideal. Nonetheless, we evaluated our database with other existing CONUS and local datasets. We also performed qualitative validation of 2015 data with the help of local experts and high resolution satellite data such as QuickBird and IKONOS. For all years, we divided the entire area into regularly spaced grids and checked each grid visually to identify and correct gross errors in the classified maps. This measure helped characterize the map qualitatively and improve the overall classification while maintaining the consistency across years. For 2015, an overall accuracy of 97% with producer and user accuracies of 92% and 94% were achieved.

**Figure 7:**
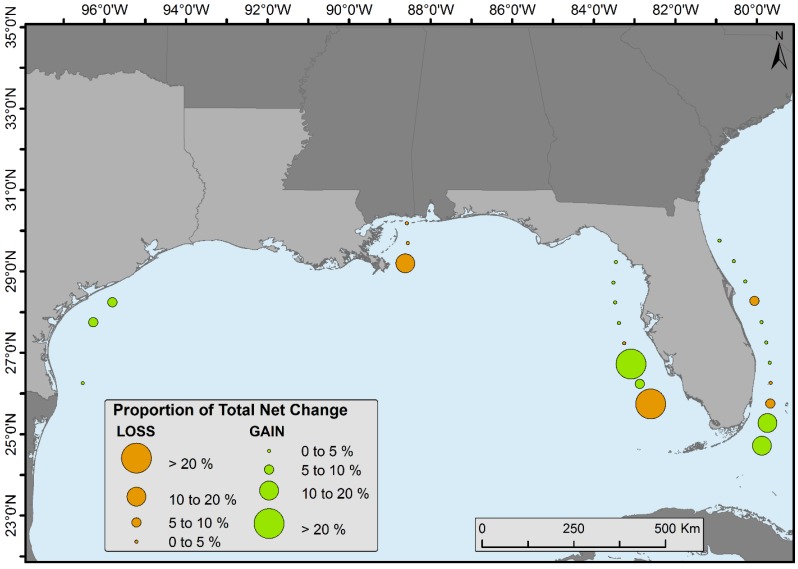
Proportion of total mangrove forest cover change per 0.5° latitudinal intervals.

Spatiotemporal change analyses were performed by dividing the CONUS mangroves into 0.5° latitudinal intervals (Figure 4). Florida was divided into halves (i.e., east and west) because of its peninsular shape (except for the bottom two zones). Proportion of forest net gain or loss was observed in all latitudinal zones (Figure 7). 

Our results revealed that the northernmost latitudinal range of mangrove along the east coast of Florida (81.317299° W, 29.945414° N) and west coast of Florida (83.046396° W, 29.162046° N), and in Louisiana (88.860357° W, 30.038007° N) and Texas (96.410255° W, 28.428913° N) has not systematically expanded toward the pole from 1980 to 2015 (Table 1). However, the historical northernmost limit before the 1980s is not well surveyed or documented and individual mangrove recruits were not mapped due to 30-m resolution of Landsat satellite data.

Relationship between (i) annual mean relative sea level rise and mangrove area change and (ii) annual minimum temperature and mangrove area change are presented in Appendix A and Appendix B, respectively.

The net mangrove increase from 1980 to 2015 is attributed to landward expansion within the northernmost limit rather than poleward migration as previously thought. Satellite observations from 1980 to 2015 do not provide evidence of poleward expansion in all three states (Table 1). For example, in Louisiana (the northernmost mangrove observed during the entire study period), the northern boundary has moved south from 1980 to 2015. This is because the forest has not recovered its previous extent after the devastating damage due to the winter freeze of 1983. On the west coast of Florida and in Texas, observed changes are negligible. The northernmost boundary along the east coast of Florida moved northward by approximately 8 km from 1980 to 2015. This observed northward shift, represented by sporadic patches of mangroves in 2015, could be highly variable and short-lived. Periodic fluctuation of ecosystem boundaries is common in many ecosystems around the world [31]. Future monitoring will tell if this shift is permanent expansion. 

The northern limit of mangrove forests in CONUS before the 1980s is not fully understood nor precisely surveyed. We did not use Landsat Multispectral Scanner (MSS) data from the 1980s or earlier because these data are available at nominal 60-meter spatial resolution with poor radiometric quantization (6 bit), thus making it difficult to discern small patches of mangrove forests in this northernmost boundary. We attempted to acquire aerial photographs from before the 1980s, but either data availability was sporadic or mangrove detection was not possible thus making it impractical to delineate the previous extent of the forest. Historical evidence suggests that a Spanish expedition led by Alvardo Mexia noted mangroves along their route from St. Augustine to the Indian River inlet in 1605. Similarly, J. R. Motte, an Army surgeon during the Seminole Wars, noted dead mangroves killed by the severe winter of 1835 in the Indian River Lagoon during the winter of 1837–1838 [32]. Savage [33] reported that mangrove distribution extended up to 30.0° N latitude in eastern Florida, and Louisiana had mangrove stands at least 75 years ago.

Surprisingly, most recent accounts of poleward migration remain anecdotal. Williams et al. [34] conducted a field survey travelling on foot and recorded a single black mangrove tree at 30.11° N and 81.37° W. It is possible that individual mangrove trees may have been unnoticed in the past, and it is not possible to discern individual trees from the 30-m Landsat satellite data used in this study. Therefore, our discussion herein targets systematic mangrove forest movement, and would necessarily exclude individual trees. Furthermore, a stable mangrove forest stand may be needed for classification as a productive and healthy forest. Other studies reported the dramatic southward expansion of the northernmost boundaries in Indian River Lagoon (IRL) [11] and in Tampa Bay [35]. Model results also showed the possibility of poleward expansion in the future [8].

**Figure 8:**
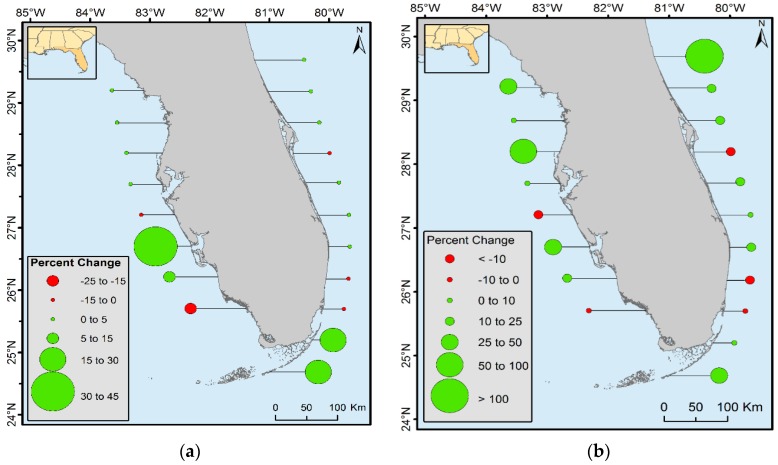
Percent of mangrove area change (**a**) percent of area change compared to total forest area of Florida and (**b**) percent of area change in each 0.5 × 0.5 degree latitudinal zone.

The landward expansion and oscillation of mangrove within the northernmost boundary were observed in CONUS. From 1980 to 2015, mangrove forest area increased in all latitudinal ranges except in the 25° to 26° latitudinal zones along both the east and west coast of Florida, 27° to 27.5° latitudinal zones on the west coast of Florida, and 28° to 28.5° latitudinal zones on the east coast of Florida. Mangrove decreased in all three latitudinal zones of Louisiana. Percent of forest increased near the northern range limit of CONUS (Figure 8b). For example, the spatial extent of mangrove between 29.5° and 30° N latitude from 1980 to 2015 doubled; however, the percent of area change in this case is misleading. An increase of >100% in this latitudinal range represents only 2% of total mangrove areal net change and 0.08% of the total mangrove area of Florida. Similarly, the northernmost boundary for the west coast of Florida changed by 25%–50%, but the total change area is only 61 ha (0.01% of Florida’s total mangrove area in 2015). Percent changes in terms of total area change in Florida are presented in Figure 8a. This figure shows that the major increase in mangrove area occurred in the southern latitudinal ranges of Florida where landward expansion is most prevalent.

**Figure 9:**
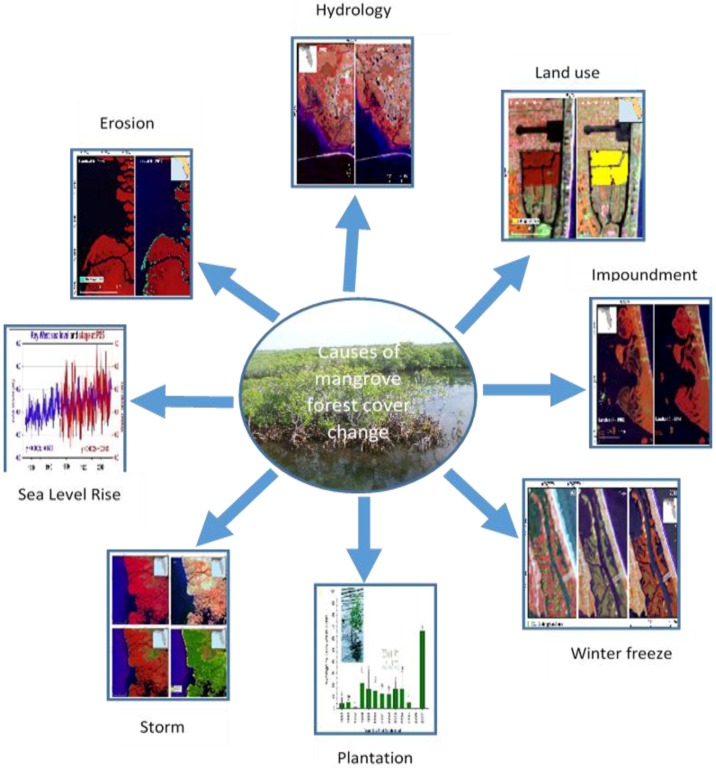
Major causes of mangrove forest cover change in CONUS.

Multiple factors are often responsible for mangrove change (Figure 9) and are site specific. In the IRL of eastern Florida, landward expansion of mangroves has been observed since the 1980s. Relatively mild winters since 1984 have likely contributed to the expansion, but a principal driver of mangrove expansion has been a result of converting closed mosquito impoundments to Rotational Impoundment Management (RIM) impoundments and restoration of dragline ditches. Impoundments built during the 1940–1960s cut off tidal circulation with long-term flooding that killed 76% of mangrove forests in the IRL [36,37]. RIM techniques were developed and implemented in the 1980s, allowing tidal water to cycle naturally into the impounded mangrove forests on a rotational basis. Similarly, during the 1950s and 1960s primarily along the central east coast of Florida, dredging of estuarine sediments and depositing them onto coastal wetlands was a common practice. In the most extensively ditched areas, up to 80% of wetlands were replaced with ditches and spoil piles. Rehabilitation of dragline ditch-impacted coastal wetlands was initiated in 1999. 

**Figure 10:**
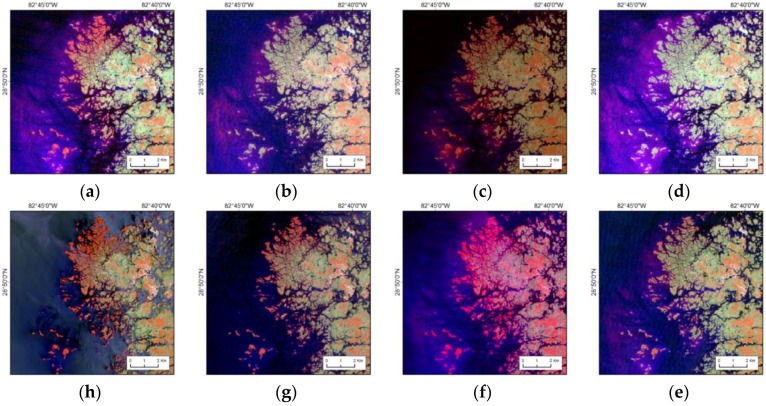
Mangrove damage due to severe winter freeze and subsequent recovery in Cedar Keys, Florida. The image is displaced in Landsat False Color Composite; dark red is mangrove forests, blue is waterbodies, and other colors represent non-mangrove and non-water. (**a**) Landsat 1982 (**b**) Landsat 1984 (**c**) Landsat 1988 (**d**) Landsat 1990 (**e**) Landsat 1995 (**f**) Landsat 2008 (**g**) Landsat 2010 (**h**) Landsat 2014.

Damage from major winter freezes of 1983, 1989, and 2010 was variable, but mangrove areas in all of the 0.5° latitudinal zones recovered to >90% of previous levels within 5–10 years (Figure 10).

For the Ten Thousand Islands, landward migration of mangrove was prominent. Detailed investigation by this study and a study by Krauss et al. [16] revealed that the change is due to compounding factors: relative sea level rise, construction of water ways, localized rainfall patterns, and reduced volume of freshwater delivery. The construction of waterways facilitated dispersal of propagules needed for natural regeneration. Similarly, a complex and disparate nature of mangrove change was observed in three adjacent sites within the Everglades [20]. Smith et al. [20] analyzed aerial photographs and images from 1928 to 2004 and found that fire assisted in mangrove expansion into salt marshes in one of the sites, whereas no change was measured in another fire-impacted site. In the third site, mangrove expansion was observed where no significant fire occurred. At this site, sea level rise was found to be the leading cause of mangrove expansion.

**Figure 11:**
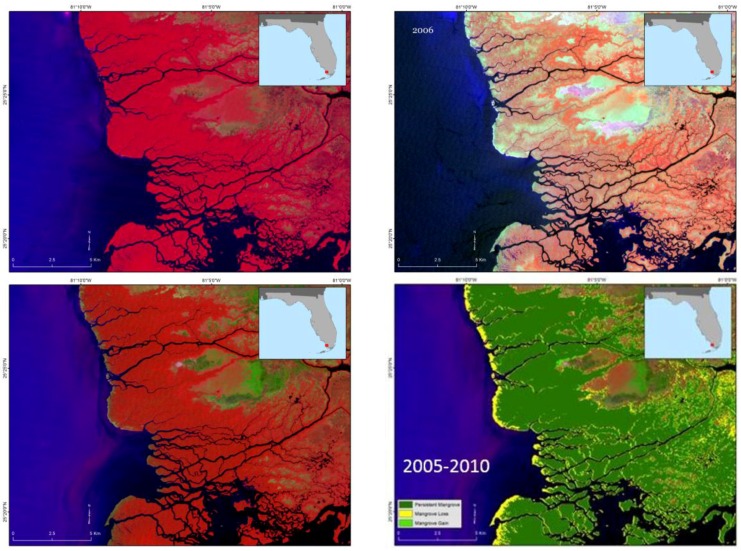
Mangrove damage due to hurricane Wilma in the winter of 2005 (**upper right**) and recovery. Much of the mangrove forest recovered except along the coastal fringes as seen in the change map from 2005–2010 (**lower right**). Landsat images are shown in False Color Composite, and dark red roughly corresponds to healthy mangrove vegetation. (**upper left**) Landsat 2005 before storm damage (**lower left**) Landsat 2010.

Mangrove forests in southern Florida are exposed to frequent tropical storms and hurricanes. For example, the passage of Hurricane Andrew across the Shark River in 1992, and Hurricane Wilma again in 2005, damaged much of the mangrove forests along Shark River, Everglades National Park. Mangrove was reestablished in much of the areas except coastal fringes by 2001 after Hurricane Andrew, and as depicted here, by 2010 after Hurricane Wilma (Figure 11). A recent satellite image of 2015 showed complete recovery of all the mangrove forests. Dead individual trees and woody debris are present on site, but the forests have visually recovered from remote sensing metrics. 

Other causes of changes include erosion, changes in hydrology, land use change, and mangrove die-offs. For example, mangrove die-off was observed at or near the J. N. Ding Darling National Wildlife Refuge in 2007.

**Figure 12:**
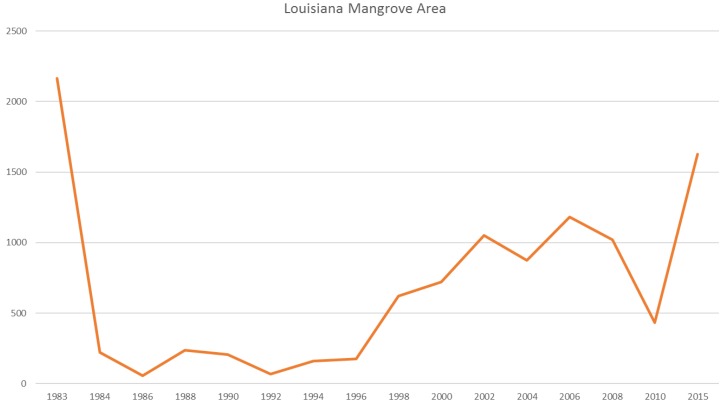
Louisiana mangrove area from 1982 to 2015.

In Louisiana, major changes occurred in the northernmost boundary from 29° to 30.5° latitude (Figure 7). Giri et al. [6] illustrated that a severe freeze in late December of 1983 reduced Louisiana’s mangrove extent by ~90%, and repeated damage from sub-freezing temperatures in 1985, 1989, and 2010 prevented mangrove extent to rebound until the late 1990s and into the 2000s. Despite less frequent winter freeze events in the past decade, Louisiana’s mangrove has not reached its historical extent (i.e., 1983) likely because of rapid coastal retrogradation and coastal [6] subsidence coupled with rapid sea level rise (9.03 mm/year) (Figure 12). Louisiana has the highest rates of relative sea level rise in the Gulf of Mexico.

**Figure 13:**
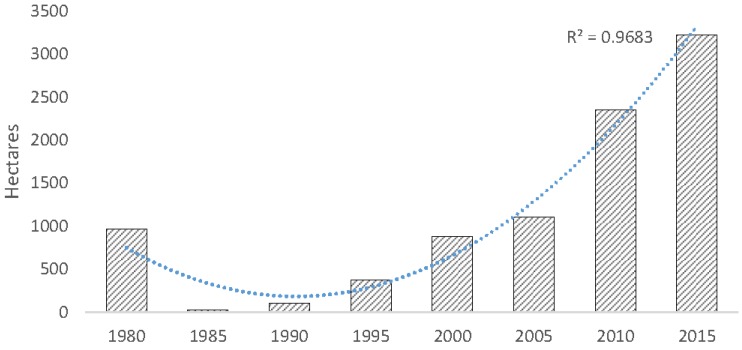
Increase of mangrove extent in Texas from 1980 to 2015.

In Texas, mangrove forests expanded significantly (Figure 13) because of the relatively mild winters, and possibly because of sea level rise. Between 1980 and 2015, mangrove area increased 2259 ha, a 234% increase. Change analysis indicated that mangrove growth was mainly at the expense of salt marshes and upland vegetation.

Major mangrove expansion was observed between 27°30′ and 28°30′ latitude. In these regions, mangrove is primarily expanding at the expense of marsh or upland. New mangrove stands emerged in the Corpus Christi Pass in the last 15 years, but mangrove has not extended more than 1 km beyond its 1980 northern range (Table 1). 

The interplay of mangrove-salt marsh ecotone could significantly alter the carbon (C) storage capacity of coastal wetland. For example, in Florida over the 7-year period (2003 to 2010), “total wetland C stocks increased 22% due to mangrove encroachment into salt marshes” [38] primarily due to differences in aboveground biomass. Similarly, the sedimentation rate of mangroves is higher than *Spartina* [38]. However, further research is needed to examine the trade-offs between mangroves and salt marshes.

Correlation coefficient between increase in mangrove areas and annual mean sea level rise, as well as annual minimum temperature in all three states revealed a stronger correlation between annual mean sea level rise and mangrove area increase in Texas (Table 2). A negative correlation in Louisiana with annual mean sea level rise and in St. Augustine with annual minimum temperature was also observed.

Model results predict expansion of mangroves at the expense of salt marshes along large areas of the Florida, Texas, and Louisiana coasts as a result of a possible increase in mean annual minimum temperature of 2–4 °C [8]. This will have important implications to climate change, wildlife, commercial and recreational fisheries, human economics, and other ecosystem goods and services.

## 4. Conclusions

We have prepared a mangrove distribution database of the entire conterminous United States (CONUS) for every five years from 1980 to 2015. Post-classification change analysis revealed that the total mangrove area of CONUS has increased from 1980 to 2015 by 4.3%. However, poleward expansion of mangroves was not observed in all three states. We observed the most substantial increases in mangrove area from 1980 to present in southern Florida and Texas within the northernmost boundaries. Major causes of mangrove changes were location specific and not universal.

While ~35 years of analysis provides reliable observations of recent drivers for range expansion and contraction, this timeframe is relatively short and the historical mangrove dynamics of the pre-satellite era remains unknown. This mangrove database could be used to monitor the future impact of climate change on mangrove forests.

## Figures and Tables

**Table 1:** Geographic coordinates of the northernmost extent of mangrove distribution in eastern Florida, western Florida, Louisiana, and Texas.

State	Latitude (in Decimal Degrees)		Longitude (in Decimal Degrees)		Distance Shift (in km)
	1980	2015	1980	2015	
Eastern Florida	29.86373	29.94541	−81.30328	−81.3173	8.0 North
Western Florida	29.16205	29.16232	−83.0464	−83.04648	0.04 North
Louisiana	30.03801	29.97985	−88.86036	−88.83519	6.3 South
Texas	28.42891	28.43685	−96.41026	−96.4012	0.88 North

**Table 2:** Linear Correlation coefficient (r) between increase in mangrove areas and annual mean sea level rise and annual minimum temperature from 1980 to 2015.

State/Place	Linear Correlation Coefficient	
	Annual Mean Sea Level Rise & Mangrove Increase	Annual Minimum Temperature & Mangrove Increase
Texas	0.688456	0.081915
Louisiana	−0.15458	0.334707
St. Augustine	0.307575	−0.50143

**Figure A1:**
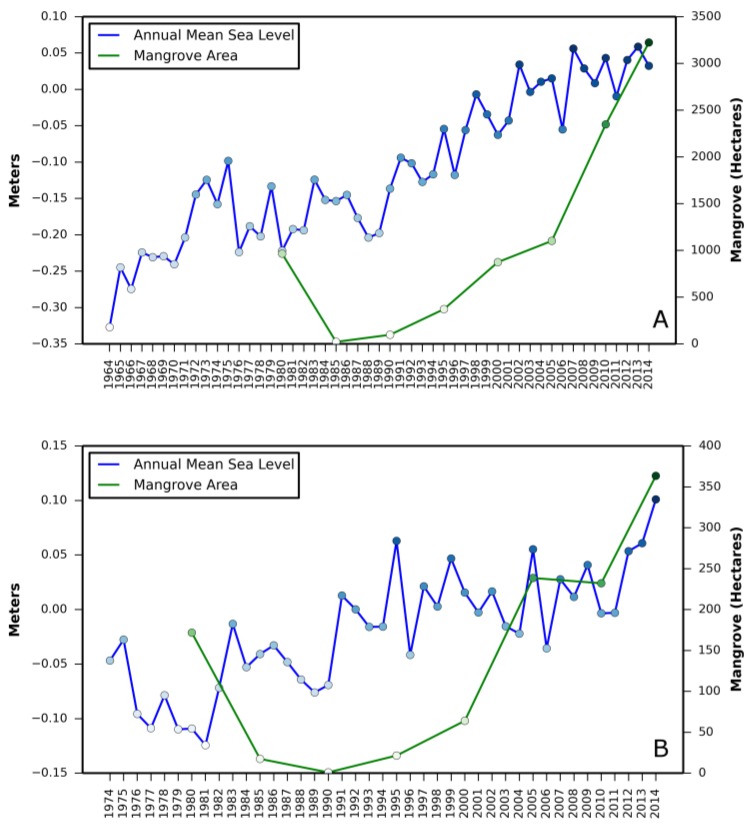

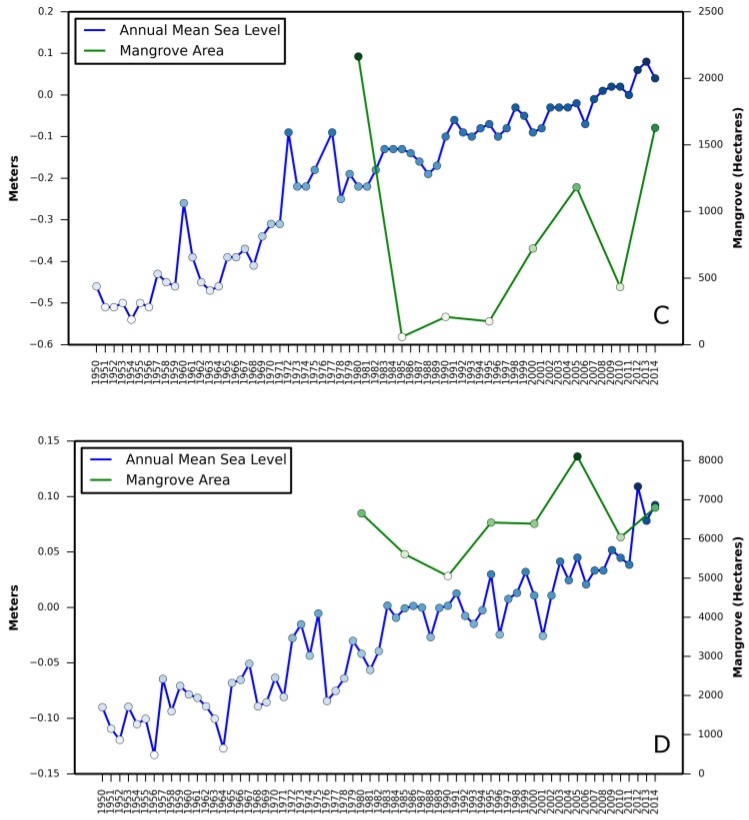
Annual mean sea level rise in (**A**) Corpus Christi, TX, USA; (**B**) St. Augustine, FL, USA; (**C**) Grand Isle, LA, USA; and (**D**) Tampa Bay, FL, USA.

**Figure B1:**
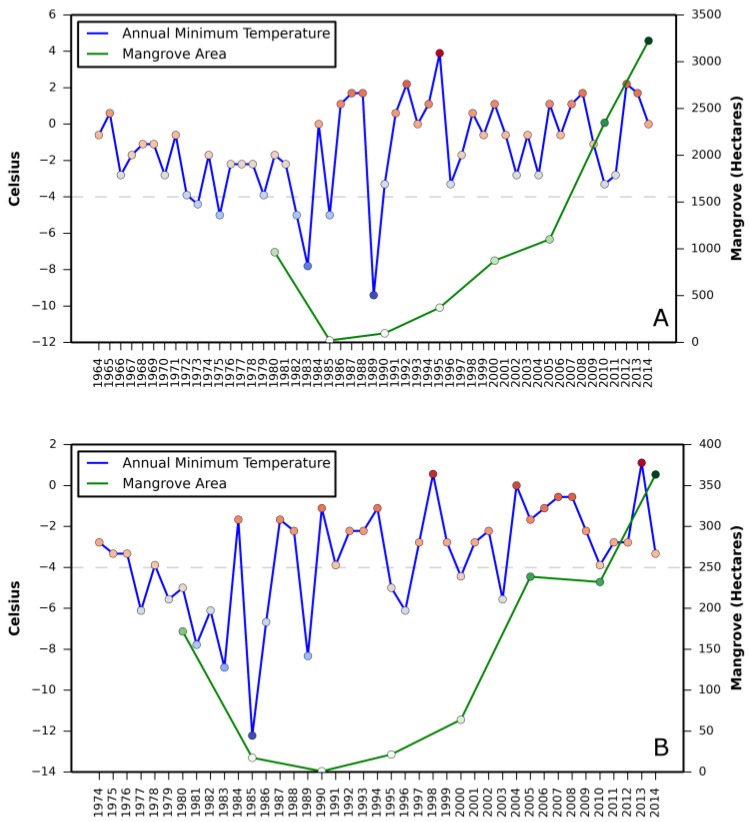

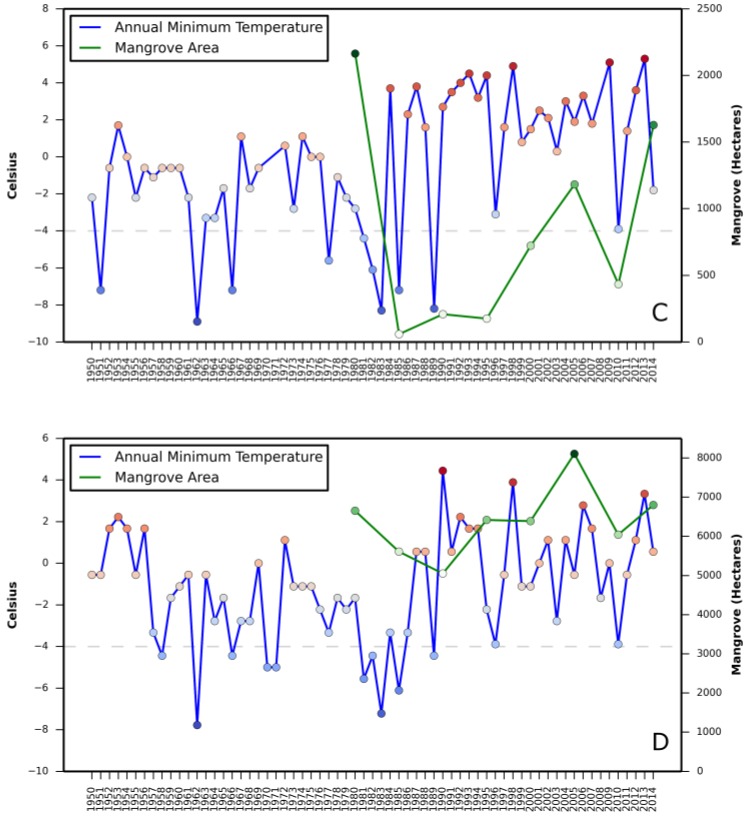
Annual minimum temperature in (**A**) Corpus Christi, TX, USA; (**B**) St. Augustine, FL, USA; (**C**) Grand Isle, LA, USA; and (**D**) Tampa Bay, FL, USA.
